# Bis[1,3-bis­(1-methyl-1*H*-benzimidazol-2-yl)-2-oxapropane]­cadmium dipicrate acetonitrile sesquisolvate 0.25-hydrate

**DOI:** 10.1107/S160053681101525X

**Published:** 2011-04-29

**Authors:** Huilu Wu, Fan Kou, Fei Jia, Jingkun Yuan, Bin Liu

**Affiliations:** a School of Chemical and Biological Engineering, Lanzhou Jiaotong University, Lanzhou 730070, People’s Republic of China

## Abstract

In the title compound, [Cd(C_18_H_18_N_4_O)_2_](C_6_H_2_N_3_O_7_)_2_·1.5CH_3_CN·0.25H_2_O, the Cd^II^ ion is coordinated by four N atoms and two O atoms from two tridentate 1,3-bis­(1-methyl-1*H*-benzimidazol-2-yl)-2-oxopropane ligands in a distorted octa­hedral coordination environment. The lengths of the chemically equivalent Cd—O bonds [2.4850 (16) and 2.5488 (16)Å] are signiificantly different. One of the picrate anions is disordered over two sets of sites, with refined occupancies of 0.504 (15) and 0.496 (15). A 0.5-occupancy acetonitrile solvent mol­ecule is disordered over two sites with equal occupancies. The H atoms of a 0.25-occupancy solvent water mol­ecule were neither located nor included in the refinement.

## Related literature

For related structures, see: Addison *et al.* (1983 ▶); Cheng *et al.* (2004 ▶); Wu *et al.* (2009*a* ▶,*b* ▶); Yun *et al.* (2008 ▶).
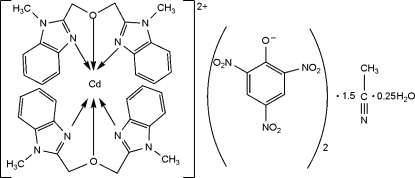


## Experimental

### 

#### Crystal data

[Cd(C_18_H_18_N_4_O)_2_](C_6_H_2_N_3_O_7_)_2_·1.5C_2_H_3_N·0.25H_2_O
*M* *_r_* = 1247.43
Monoclinic, 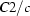

*a* = 28.5192 (5) Å
*b* = 18.1631 (3) Å
*c* = 25.4194 (5) Å
β = 122.697 (1)°
*V* = 11080.7 (3) Å^3^
*Z* = 8
Mo *K*α radiation
μ = 0.48 mm^−1^
*T* = 153 K
0.58 × 0.54 × 0.42 mm

#### Data collection

Rigaku R-AXIS Spider diffractometer
Absorption correction: multi-scan (*ABSCOR*; Higashi, 1995 ▶) *T*_min_ = 0.862, *T*_max_ = 0.898
51747 measured reflections
12631 independent reflections
11048 reflections with *I* > 2σ(*I*)
*R*_int_ = 0.023

#### Refinement

*R*[*F* ^2^ > 2σ(*F* ^2^)] = 0.034
*wR*(*F* ^2^) = 0.101
*S* = 1.05
12631 reflections
813 parameters
48 restraints
H-atom parameters constrained
Δρ_max_ = 1.14 e Å^−3^
Δρ_min_ = −0.85 e Å^−3^

### 

Data collection: *RAPID-AUTO* (Rigaku/MSC, 2004) ▶; cell refinement: *RAPID-AUTO* ▶; data reduction: *RAPID-AUTO* ▶; program(s) used to solve structure: *SHELXS97* (Sheldrick, 2008 ▶); program(s) used to refine structure: *SHELXL97* (Sheldrick, 2008 ▶); molecular graphics: *SHELXTL* (Sheldrick, 2008 ▶); software used to prepare material for publication: *SHELXTL*.

## Supplementary Material

Crystal structure: contains datablocks global, I. DOI: 10.1107/S160053681101525X/lh5236sup1.cif

Structure factors: contains datablocks I. DOI: 10.1107/S160053681101525X/lh5236Isup2.hkl

Additional supplementary materials:  crystallographic information; 3D view; checkCIF report

## Figures and Tables

**Fig. 1.**
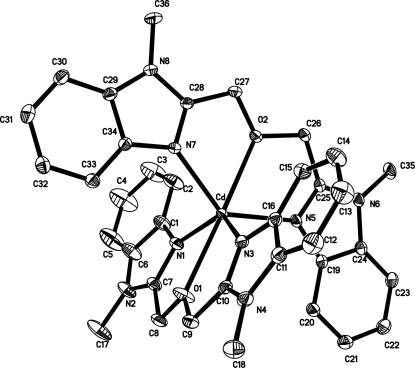
The molecular structure of the cation. Hydrogen atoms have been omitted for clarity and the displacement ellipsoids are shown at the 30% probability level.

## supplementary crystallographic information

### Comment

Interest in bis(2-benzimidazolyl)alkanes and their derivatives is widespread (Addison *et al.*, 1983; Cheng *et al.*, 2004). We have previously reported the crystal structures of some related complexes (Wu *et al.*, 2009*a*,*b*; Yun *et al.*, 2008). The asymmetric unit of the title compound, consists of a discrete [Cd(meobb)_2_] cation (Fig. 1), two picrate anions, 1.5 molecules of acetonitrile and one quarter water molecule. The cadmium ion is six-coordinate with a N_4_O_2_ ligand set. The meobb ligand acts as a tridentate N-donor and O-donor. The coordination geometry of the Cd^II^ ion may be best described as distorted octahedral with four coordination nitrogen atoms distorted from an ideal equatorial plane. The axial sites are occupyied by O1 and O2. The O atoms can be considered as weakly coordinated. In the crystal the shortest π···π stacking distance is 3.875 (3)Å.

### Experimental

To a stirred solution of meobb (0.153 g, 0.5 mmol) in hot MeOH (15 ml) was added Cd(C_6_H_2_N_3_O_7_)_2_ (0.153 g, 0.25 mmol) in MeOH (5 ml). A yellow crystalline product formed rapidly. The precipitate was filtered off, washed with MeOH and absolute Et_2_O, and dried *in vacuo*. The dried precipitate was dissolved in acetonitrile to a yellow solution that was allowed to evaporate at room temperature. The yellow crystals suitable for X-ray diffraction studies were obtained after three weeks. Yield, 0.229 g (75%).

### Refinement

All H atoms were found in difference electron maps and were subsequently refined in a riding-model approximation with C—H distances ranging from 0.95 to 0.99 Å and *U*_iso_(H) = 1.2 *U*_eq_(C) or 1.5 *U*_eq_(C_methyl_). One of the picrate anions is disordered over two sets of sites with refined occupancies 0.504 (15) and 0.496 (15). A 0.5 occupancy acetonitrile solvent molecule is disrodered over two sites with equall occupancy. The H atoms of a 0.25 occupancy solvent water molecule were not located nor included in the refinement but the H atoms are included in the formula.

### Crystal data

**Table:**

[Cd(C_18_H_18_N_4_O)_2_](C_6_H_2_N_3_O_7_)_2_·1.5C_2_H_3_N·0.25H_2_O	*F*(000) = 5100
*M**_r_* = 1247.43	*D*_x_ = 1.496 Mg m^−^^3^
Monoclinic, *C*2/*c*	Mo *K*α radiation, λ = 0.71073 Å
Hall symbol: -C 2yc	Cell parameters from 7749 reflections
*a* = 28.5192 (5) Å	θ = 3.2–27.5°
*b* = 18.1631 (3) Å	µ = 0.48 mm^−^^1^
*c* = 25.4194 (5) Å	*T* = 153 K
β = 122.697 (1)°	Block, yellow
*V* = 11080.7 (3) Å^3^	0.58 × 0.54 × 0.42 mm
*Z* = 8	

### Data collection

**Table:**

Rigaku R-AXIS Spider diffractometer	12631 independent reflections
Radiation source: fine-focus sealed tube	11048 reflections with *I* > 2σ(*I*)
graphite	*R*_int_ = 0.023
φ and ω scans	θ_max_ = 27.5°, θ_min_ = 3.2°
Absorption correction: multi-scan (*ABSCOR*; Higashi, 1995)	*h* = −36→36
*T*_min_ = 0.862, *T*_max_ = 0.898	*k* = −23→23
51747 measured reflections	*l* = −32→33

### Refinement

**Table:**

Refinement on *F*^2^	Primary atom site location: structure-invariant direct methods
Least-squares matrix: full	Secondary atom site location: difference Fourier map
*R*[*F*^2^ > 2σ(*F*^2^)] = 0.034	Hydrogen site location: inferred from neighbouring sites
*wR*(*F*^2^) = 0.101	H-atom parameters constrained
*S* = 1.05	*w* = 1/[σ^2^(*F*_o_^2^) + (0.0565*P*)^2^ + 18.4728*P*] where *P* = (*F*_o_^2^ + 2*F*_c_^2^)/3
12631 reflections	(Δ/σ)_max_ = 0.001
813 parameters	Δρ_max_ = 1.14 e Å^−^^3^
48 restraints	Δρ_min_ = −0.85 e Å^−^^3^

### Special details

**Table:**

Geometry. All esds (except the esd in the dihedral angle between two l.s. planes) are estimated using the full covariance matrix. The cell esds are taken into account individually in the estimation of esds in distances, angles and torsion angles; correlations between esds in cell parameters are only used when they are defined by crystal symmetry. An approximate (isotropic) treatment of cell esds is used for estimating esds involving l.s. planes.
Refinement. Refinement of F^2^ against ALL reflections. The weighted R-factor wR and goodness of fit S are based on F^2^, conventional R-factors R are based on F, with F set to zero for negative F^2^. The threshold expression of F^2^ > 2sigma(F^2^) is used only for calculating R-factors(gt) etc. and is not relevant to the choice of reflections for refinement. R-factors based on F^2^ are statistically about twice as large as those based on F, and R- factors based on ALL data will be even larger.

### Fractional atomic coordinates and isotropic or equivalent isotropic displacement parameters (Å^2^)

**Table:**

	*x*	*y*	*z*	*U*_iso_*/*U*_eq_	Occ. (<1)
Cd	0.299484 (6)	0.121031 (7)	0.330165 (6)	0.01844 (6)	
O1	0.26484 (7)	0.06428 (12)	0.39188 (8)	0.0419 (5)	
O2	0.34653 (9)	0.18207 (9)	0.28007 (8)	0.0366 (4)	
N1	0.36649 (8)	0.10160 (10)	0.43043 (8)	0.0258 (4)	
N2	0.40481 (10)	0.05758 (13)	0.52649 (9)	0.0389 (5)	
N3	0.20723 (7)	0.09654 (9)	0.27406 (8)	0.0218 (3)	
N4	0.12489 (8)	0.06046 (10)	0.25523 (9)	0.0277 (4)	
N5	0.29001 (7)	0.24327 (9)	0.32272 (8)	0.0213 (3)	
N6	0.30453 (8)	0.35829 (10)	0.30522 (9)	0.0241 (4)	
N7	0.33174 (7)	0.04057 (9)	0.29023 (8)	0.0208 (3)	
N8	0.37633 (8)	0.00252 (10)	0.24547 (8)	0.0240 (4)	
C1	0.42397 (11)	0.10573 (15)	0.46039 (11)	0.0345 (5)	
C2	0.45674 (12)	0.13093 (18)	0.43868 (13)	0.0460 (7)	
H2A	0.4404	0.1502	0.3976	0.055*	
C3	0.51350 (14)	0.1268 (3)	0.47890 (16)	0.0723 (12)	
H3A	0.5369	0.1442	0.4656	0.087*	
C4	0.53767 (15)	0.0976 (3)	0.53926 (17)	0.0876 (15)	
H4A	0.5771	0.0946	0.5656	0.105*	
C5	0.50572 (14)	0.0730 (3)	0.56147 (14)	0.0727 (12)	
H5A	0.5221	0.0535	0.6025	0.087*	
C6	0.44846 (12)	0.07813 (18)	0.52082 (12)	0.0453 (7)	
C7	0.35718 (10)	0.07241 (12)	0.47135 (10)	0.0281 (5)	
C8	0.30009 (10)	0.05688 (14)	0.45734 (10)	0.0309 (5)	
H8A	0.2979	0.0065	0.4707	0.037*	
H8B	0.2898	0.0926	0.4789	0.037*	
C9	0.20837 (10)	0.04559 (13)	0.36501 (11)	0.0297 (5)	
H9A	0.1925	0.0727	0.3857	0.036*	
H9B	0.2043	−0.0079	0.3689	0.036*	
C10	0.17993 (9)	0.06741 (11)	0.29774 (10)	0.0235 (4)	
C11	0.11516 (9)	0.08786 (12)	0.19891 (11)	0.0272 (4)	
C12	0.06697 (11)	0.09476 (16)	0.13996 (13)	0.0400 (6)	
H12A	0.0317	0.0795	0.1316	0.048*	
C13	0.07300 (12)	0.12492 (17)	0.09413 (14)	0.0460 (7)	
H13A	0.0409	0.1312	0.0533	0.055*	
C14	0.12501 (11)	0.14665 (16)	0.10585 (12)	0.0382 (6)	
H14A	0.1273	0.1669	0.0728	0.046*	
C15	0.17307 (10)	0.13914 (13)	0.16449 (10)	0.0283 (5)	
H15A	0.2084	0.1532	0.1725	0.034*	
C16	0.16720 (9)	0.10992 (11)	0.21151 (10)	0.0228 (4)	
C17	0.40956 (15)	0.0278 (2)	0.58261 (12)	0.0626 (10)	
H17A	0.3725	0.0138	0.5730	0.094*	
H17B	0.4255	0.0652	0.6156	0.094*	
H17C	0.4338	−0.0156	0.5969	0.094*	
C18	0.08242 (11)	0.02886 (16)	0.26413 (15)	0.0430 (6)	
H18A	0.0991	0.0170	0.3084	0.065*	
H18B	0.0674	−0.0161	0.2391	0.065*	
H18C	0.0522	0.0645	0.2508	0.065*	
C19	0.27055 (9)	0.29043 (11)	0.34982 (10)	0.0218 (4)	
C20	0.24570 (10)	0.27499 (12)	0.38291 (10)	0.0269 (4)	
H20A	0.2394	0.2258	0.3902	0.032*	
C21	0.23058 (11)	0.33411 (13)	0.40476 (12)	0.0335 (5)	
H21A	0.2131	0.3254	0.4271	0.040*	
C22	0.24056 (11)	0.40694 (13)	0.39450 (12)	0.0348 (5)	
H22A	0.2300	0.4463	0.4106	0.042*	
C23	0.26518 (10)	0.42301 (12)	0.36175 (11)	0.0303 (5)	
H23A	0.2719	0.4723	0.3549	0.036*	
C24	0.27965 (9)	0.36313 (12)	0.33926 (10)	0.0238 (4)	
C25	0.30962 (9)	0.28560 (11)	0.29698 (10)	0.0216 (4)	
C26	0.33478 (10)	0.25727 (11)	0.26257 (10)	0.0250 (4)	
H26A	0.3083	0.2621	0.2169	0.030*	
H26B	0.3693	0.2845	0.2749	0.030*	
C27	0.35949 (10)	0.13814 (12)	0.24365 (10)	0.0256 (4)	
H27A	0.3975	0.1492	0.2538	0.031*	
H27B	0.3326	0.1472	0.1985	0.031*	
C28	0.35576 (9)	0.06047 (11)	0.25997 (9)	0.0215 (4)	
C29	0.36456 (9)	−0.06028 (12)	0.26718 (10)	0.0239 (4)	
C30	0.37405 (10)	−0.13480 (13)	0.26362 (11)	0.0314 (5)	
H30A	0.3924	−0.1515	0.2439	0.038*	
C31	0.35529 (11)	−0.18329 (13)	0.29043 (12)	0.0349 (5)	
H31A	0.3607	−0.2346	0.2886	0.042*	
C32	0.32875 (10)	−0.15936 (13)	0.32001 (11)	0.0304 (5)	
H32A	0.3170	−0.1945	0.3382	0.036*	
C33	0.31914 (9)	−0.08489 (12)	0.32339 (10)	0.0253 (4)	
H33A	0.3011	−0.0684	0.3436	0.030*	
C34	0.33698 (8)	−0.03589 (11)	0.29598 (9)	0.0213 (4)	
C35	0.32305 (11)	0.41939 (13)	0.28366 (13)	0.0344 (5)	
H35A	0.3231	0.4041	0.2467	0.052*	
H35B	0.3608	0.4340	0.3169	0.052*	
H35C	0.2977	0.4612	0.2728	0.052*	
C36	0.40395 (12)	0.00594 (15)	0.21100 (13)	0.0373 (6)	
H36A	0.4312	0.0461	0.2276	0.056*	
H36B	0.3761	0.0148	0.1666	0.056*	
H36C	0.4230	−0.0408	0.2157	0.056*	
O3	0.1456 (3)	−0.0423 (3)	−0.0743 (4)	0.0484 (17)	0.496 (15)
O4	0.0579 (7)	0.0579 (6)	−0.1197 (3)	0.053 (3)	0.496 (15)
O5	0.0860 (8)	0.1102 (6)	−0.0306 (6)	0.0451 (19)	0.496 (15)
O6	0.0433 (15)	−0.0804 (11)	0.0835 (16)	0.046 (4)	0.496 (15)
O7	0.0783 (13)	−0.1894 (9)	0.0952 (12)	0.040 (3)	0.496 (15)
O8	0.1464 (6)	−0.2605 (4)	−0.0310 (7)	0.0414 (16)	0.496 (15)
O9	0.1891 (5)	−0.1795 (7)	−0.0539 (7)	0.050 (2)	0.496 (15)
N9	0.0767 (2)	0.0560 (3)	−0.0632 (3)	0.0299 (8)	0.496 (15)
N10	0.0634 (4)	−0.1293 (4)	0.0676 (4)	0.0299 (8)	0.496 (15)
N11	0.1580 (3)	−0.1959 (4)	−0.0368 (2)	0.0299 (8)	0.496 (15)
C37	0.1216 (2)	−0.0664 (3)	−0.0467 (2)	0.0299 (8)	0.496 (15)
C38	0.0914 (2)	−0.0170 (2)	−0.0346 (2)	0.0299 (8)	0.496 (15)
C39	0.0701 (2)	−0.0389 (3)	0.0009 (3)	0.0299 (8)	0.496 (15)
H39	0.0494	−0.0051	0.0092	0.036*	0.496 (15)
C40	0.0791 (3)	−0.1102 (3)	0.0243 (3)	0.0299 (8)	0.496 (15)
C41	0.1093 (2)	−0.1596 (2)	0.0122 (2)	0.0299 (8)	0.496 (15)
H41	0.1155	−0.2084	0.0282	0.036*	0.496 (15)
C42	0.1306 (2)	−0.1377 (3)	−0.0233 (2)	0.0299 (8)	0.496 (15)
O3A	0.1227 (5)	−0.0741 (8)	−0.1003 (5)	0.087 (4)	0.504 (15)
O4A	0.0451 (7)	0.0393 (7)	−0.1321 (3)	0.059 (3)	0.504 (15)
O5A	0.0821 (8)	0.0948 (5)	−0.0427 (5)	0.052 (2)	0.504 (15)
O6A	0.0358 (15)	−0.0751 (10)	0.0787 (15)	0.043 (3)	0.504 (15)
O7A	0.0684 (13)	−0.1862 (8)	0.0924 (13)	0.040 (4)	0.504 (15)
O8A	0.1405 (7)	−0.2802 (5)	−0.0274 (8)	0.060 (2)	0.504 (15)
O9A	0.1788 (6)	−0.1971 (7)	−0.0553 (8)	0.062 (3)	0.504 (15)
N9A	0.0702 (2)	0.0394 (4)	−0.0750 (3)	0.0323 (8)	0.504 (15)
N10A	0.0632 (4)	−0.1225 (4)	0.0721 (4)	0.0323 (8)	0.504 (15)
N11A	0.1470 (3)	−0.2158 (5)	−0.0386 (2)	0.0323 (8)	0.504 (15)
C37A	0.1105 (3)	−0.0869 (4)	−0.0608 (3)	0.0323 (8)	0.504 (15)
C38A	0.0834 (2)	−0.0324 (4)	−0.0438 (3)	0.0323 (8)	0.504 (15)
C39A	0.0712 (3)	−0.0402 (4)	0.0017 (3)	0.0323 (8)	0.504 (15)
H39A	0.0564	−0.0007	0.0128	0.039*	0.504 (15)
C40A	0.0817 (3)	−0.1087 (4)	0.0304 (3)	0.0323 (8)	0.504 (15)
C41A	0.1038 (3)	−0.1670 (3)	0.0145 (3)	0.0323 (8)	0.504 (15)
H41A	0.1077	−0.2146	0.0320	0.039*	0.504 (15)
C42A	0.1199 (2)	−0.1543 (4)	−0.0273 (2)	0.0323 (8)	0.504 (15)
O10	0.25476 (7)	0.72895 (9)	0.38655 (7)	0.0331 (4)	
O11	0.23974 (9)	0.58200 (10)	0.38755 (8)	0.0403 (4)	
O12	0.21537 (8)	0.56317 (10)	0.45331 (9)	0.0420 (4)	
O13	0.35914 (9)	0.63635 (11)	0.66491 (8)	0.0412 (4)	
O14	0.39345 (8)	0.74662 (11)	0.67826 (8)	0.0406 (4)	
O15	0.36237 (9)	0.88401 (10)	0.49750 (11)	0.0489 (5)	
O16	0.27765 (8)	0.87300 (9)	0.41825 (8)	0.0360 (4)	
N12	0.24252 (8)	0.59618 (10)	0.43636 (9)	0.0286 (4)	
N13	0.36553 (8)	0.69456 (12)	0.64433 (9)	0.0307 (4)	
N14	0.31871 (8)	0.84920 (11)	0.46653 (10)	0.0299 (4)	
C43	0.28021 (9)	0.72150 (12)	0.44401 (10)	0.0239 (4)	
C44	0.27884 (9)	0.65514 (11)	0.47564 (10)	0.0234 (4)	
C45	0.30566 (9)	0.64627 (12)	0.53892 (10)	0.0248 (4)	
H45	0.3018	0.6019	0.5560	0.030*	
C46	0.33859 (9)	0.70308 (12)	0.57785 (10)	0.0248 (4)	
C47	0.34390 (9)	0.76879 (12)	0.55323 (10)	0.0253 (4)	
H47	0.3671	0.8071	0.5802	0.030*	
C48	0.31512 (9)	0.77740 (11)	0.48950 (10)	0.0230 (4)	
N15	0.51231 (16)	0.1314 (2)	0.32991 (17)	0.0788 (10)	
C49	0.50121 (15)	0.2701 (2)	0.33945 (19)	0.0720 (11)	
H49A	0.4856	0.2933	0.2985	0.108*	
H49B	0.5376	0.2920	0.3694	0.108*	
H49C	0.4760	0.2779	0.3539	0.108*	
C50	0.50764 (13)	0.1936 (2)	0.33446 (14)	0.0574 (9)	
N17	0.0073 (4)	−0.2706 (4)	−0.1301 (4)	0.0413 (10)	0.25
C51	0.0233 (4)	−0.1696 (5)	−0.1889 (5)	0.0413 (10)	0.25
H51A	0.0262	−0.1221	−0.1690	0.062*	0.25
H51B	−0.0073	−0.1678	−0.2330	0.062*	0.25
H51C	0.0583	−0.1799	−0.1861	0.062*	0.25
C52	0.0130 (5)	−0.2251 (5)	−0.1585 (5)	0.0413 (10)	0.25
N17'	0.0090 (4)	−0.3585 (4)	−0.2314 (4)	0.0413 (10)	0.25
C51'	0.0045 (5)	−0.2498 (5)	−0.1700 (6)	0.0413 (10)	0.25
H51D	0.0227	−0.2077	−0.1757	0.062*	0.25
H51E	0.0227	−0.2604	−0.1253	0.062*	0.25
H51F	−0.0349	−0.2383	−0.1876	0.062*	0.25
C52'	0.0085 (4)	−0.3119 (5)	−0.2006 (5)	0.0413 (10)	0.25
O1W	0.1049 (7)	−0.2090 (6)	−0.1928 (5)	0.099 (4)	0.25

### Atomic displacement parameters (Å^2^)

**Table:**

	*U*^11^	*U*^22^	*U*^33^	*U*^12^	*U*^13^	*U*^23^
Cd	0.02384 (9)	0.01664 (8)	0.01959 (8)	0.00003 (5)	0.01485 (7)	0.00183 (5)
O1	0.0315 (9)	0.0696 (13)	0.0264 (8)	−0.0084 (9)	0.0168 (7)	0.0127 (9)
O2	0.0670 (13)	0.0223 (8)	0.0417 (10)	0.0104 (8)	0.0433 (10)	0.0095 (7)
N1	0.0317 (10)	0.0278 (9)	0.0204 (8)	−0.0021 (7)	0.0158 (8)	0.0020 (7)
N2	0.0459 (13)	0.0478 (13)	0.0172 (9)	−0.0135 (10)	0.0132 (9)	0.0031 (9)
N3	0.0256 (9)	0.0180 (8)	0.0269 (9)	−0.0009 (7)	0.0176 (8)	−0.0008 (7)
N4	0.0252 (9)	0.0256 (9)	0.0390 (10)	−0.0008 (7)	0.0218 (9)	0.0007 (8)
N5	0.0256 (9)	0.0170 (8)	0.0228 (8)	−0.0009 (6)	0.0141 (7)	0.0024 (7)
N6	0.0263 (9)	0.0166 (8)	0.0301 (9)	−0.0003 (7)	0.0158 (8)	0.0046 (7)
N7	0.0251 (9)	0.0218 (8)	0.0186 (8)	0.0015 (7)	0.0139 (7)	0.0005 (7)
N8	0.0276 (9)	0.0265 (9)	0.0246 (9)	0.0028 (7)	0.0184 (8)	0.0000 (7)
C1	0.0331 (13)	0.0440 (13)	0.0201 (10)	−0.0081 (10)	0.0104 (10)	0.0018 (10)
C2	0.0316 (14)	0.073 (2)	0.0285 (13)	−0.0094 (13)	0.0129 (11)	0.0089 (13)
C3	0.0339 (16)	0.128 (4)	0.0405 (17)	−0.0201 (19)	0.0104 (14)	0.0163 (19)
C4	0.0329 (17)	0.157 (4)	0.0447 (19)	−0.020 (2)	0.0022 (15)	0.026 (2)
C5	0.0434 (18)	0.117 (3)	0.0285 (14)	−0.020 (2)	0.0005 (13)	0.0212 (18)
C6	0.0409 (15)	0.0613 (18)	0.0230 (11)	−0.0155 (13)	0.0101 (11)	0.0041 (12)
C7	0.0391 (13)	0.0264 (11)	0.0202 (10)	−0.0057 (9)	0.0168 (10)	−0.0014 (8)
C8	0.0426 (14)	0.0335 (12)	0.0241 (10)	−0.0036 (10)	0.0231 (10)	0.0029 (9)
C9	0.0321 (12)	0.0313 (11)	0.0348 (12)	−0.0025 (9)	0.0241 (10)	0.0031 (10)
C10	0.0274 (11)	0.0179 (9)	0.0323 (11)	0.0003 (8)	0.0208 (9)	−0.0008 (8)
C11	0.0266 (11)	0.0229 (10)	0.0352 (12)	−0.0009 (8)	0.0187 (10)	−0.0008 (9)
C12	0.0260 (12)	0.0451 (14)	0.0441 (14)	−0.0039 (10)	0.0157 (11)	−0.0025 (12)
C13	0.0319 (14)	0.0595 (18)	0.0330 (13)	−0.0036 (12)	0.0086 (11)	−0.0001 (12)
C14	0.0374 (14)	0.0451 (14)	0.0269 (12)	−0.0053 (11)	0.0140 (11)	0.0017 (11)
C15	0.0311 (12)	0.0286 (11)	0.0267 (11)	−0.0046 (9)	0.0165 (10)	−0.0030 (9)
C16	0.0254 (11)	0.0173 (9)	0.0270 (10)	−0.0004 (7)	0.0149 (9)	−0.0020 (8)
C17	0.071 (2)	0.085 (2)	0.0207 (12)	−0.0314 (19)	0.0174 (13)	0.0081 (14)
C18	0.0320 (13)	0.0472 (15)	0.0618 (17)	−0.0037 (11)	0.0332 (13)	0.0080 (13)
C19	0.0238 (10)	0.0178 (9)	0.0229 (10)	0.0004 (7)	0.0119 (8)	0.0015 (8)
C20	0.0339 (12)	0.0223 (10)	0.0288 (11)	−0.0005 (8)	0.0197 (10)	0.0018 (9)
C21	0.0434 (14)	0.0297 (12)	0.0369 (13)	0.0011 (10)	0.0278 (11)	−0.0003 (10)
C22	0.0457 (15)	0.0246 (11)	0.0400 (13)	0.0036 (10)	0.0269 (12)	−0.0041 (10)
C23	0.0376 (13)	0.0170 (10)	0.0376 (12)	0.0007 (9)	0.0211 (11)	0.0002 (9)
C24	0.0250 (11)	0.0198 (9)	0.0250 (10)	−0.0007 (8)	0.0124 (9)	0.0017 (8)
C25	0.0226 (10)	0.0184 (9)	0.0222 (9)	−0.0001 (7)	0.0110 (8)	0.0027 (8)
C26	0.0325 (11)	0.0201 (10)	0.0270 (10)	0.0003 (8)	0.0190 (9)	0.0047 (8)
C27	0.0328 (12)	0.0264 (10)	0.0265 (10)	0.0009 (9)	0.0219 (9)	0.0017 (9)
C28	0.0225 (10)	0.0249 (10)	0.0192 (9)	0.0021 (8)	0.0127 (8)	0.0006 (8)
C29	0.0248 (10)	0.0259 (10)	0.0212 (9)	0.0016 (8)	0.0126 (8)	−0.0023 (8)
C30	0.0357 (13)	0.0283 (11)	0.0331 (12)	0.0041 (9)	0.0204 (10)	−0.0040 (9)
C31	0.0389 (14)	0.0235 (11)	0.0399 (13)	0.0027 (9)	0.0198 (11)	−0.0015 (10)
C32	0.0338 (12)	0.0248 (11)	0.0290 (11)	−0.0022 (9)	0.0146 (10)	0.0015 (9)
C33	0.0261 (11)	0.0261 (11)	0.0231 (10)	−0.0009 (8)	0.0130 (9)	−0.0001 (8)
C34	0.0209 (10)	0.0231 (10)	0.0178 (9)	0.0012 (7)	0.0091 (8)	−0.0014 (8)
C35	0.0382 (13)	0.0217 (10)	0.0474 (14)	−0.0014 (9)	0.0259 (12)	0.0113 (10)
C36	0.0475 (15)	0.0414 (14)	0.0429 (14)	0.0052 (11)	0.0376 (13)	0.0005 (11)
O3	0.065 (4)	0.049 (3)	0.066 (4)	0.006 (2)	0.058 (3)	0.005 (3)
O4	0.088 (8)	0.049 (4)	0.028 (3)	0.009 (4)	0.035 (4)	0.004 (3)
O5	0.056 (5)	0.040 (4)	0.039 (4)	−0.023 (4)	0.025 (4)	−0.014 (3)
O6	0.044 (8)	0.067 (6)	0.047 (6)	0.022 (4)	0.038 (6)	0.018 (4)
O7	0.028 (8)	0.057 (5)	0.037 (4)	0.006 (3)	0.019 (4)	0.018 (3)
O8	0.061 (4)	0.022 (4)	0.058 (4)	0.006 (3)	0.043 (3)	0.012 (4)
O9	0.043 (5)	0.067 (5)	0.058 (4)	0.005 (4)	0.039 (3)	0.002 (4)
N9	0.0248 (11)	0.0396 (13)	0.0259 (12)	−0.0023 (9)	0.0141 (9)	−0.0023 (10)
N10	0.0248 (11)	0.0396 (13)	0.0259 (12)	−0.0023 (9)	0.0141 (9)	−0.0023 (10)
N11	0.0248 (11)	0.0396 (13)	0.0259 (12)	−0.0023 (9)	0.0141 (9)	−0.0023 (10)
C37	0.0248 (11)	0.0396 (13)	0.0259 (12)	−0.0023 (9)	0.0141 (9)	−0.0023 (10)
C38	0.0248 (11)	0.0396 (13)	0.0259 (12)	−0.0023 (9)	0.0141 (9)	−0.0023 (10)
C39	0.0248 (11)	0.0396 (13)	0.0259 (12)	−0.0023 (9)	0.0141 (9)	−0.0023 (10)
C40	0.0248 (11)	0.0396 (13)	0.0259 (12)	−0.0023 (9)	0.0141 (9)	−0.0023 (10)
C41	0.0248 (11)	0.0396 (13)	0.0259 (12)	−0.0023 (9)	0.0141 (9)	−0.0023 (10)
C42	0.0248 (11)	0.0396 (13)	0.0259 (12)	−0.0023 (9)	0.0141 (9)	−0.0023 (10)
O3A	0.090 (6)	0.134 (9)	0.080 (6)	0.064 (6)	0.075 (5)	0.062 (6)
O4A	0.070 (6)	0.064 (6)	0.034 (3)	0.019 (5)	0.024 (4)	0.016 (3)
O5A	0.059 (4)	0.035 (4)	0.040 (5)	−0.026 (4)	0.012 (4)	−0.002 (3)
O6A	0.043 (8)	0.053 (6)	0.050 (6)	0.012 (4)	0.036 (6)	0.010 (4)
O7A	0.031 (9)	0.046 (4)	0.053 (6)	0.008 (3)	0.028 (6)	0.018 (3)
O8A	0.087 (6)	0.036 (4)	0.076 (5)	0.011 (5)	0.055 (4)	0.013 (5)
O9A	0.061 (6)	0.081 (7)	0.080 (5)	−0.004 (4)	0.063 (5)	−0.015 (5)
N9A	0.0233 (11)	0.0483 (14)	0.0279 (12)	0.0001 (10)	0.0155 (9)	0.0043 (10)
N10A	0.0233 (11)	0.0483 (14)	0.0279 (12)	0.0001 (10)	0.0155 (9)	0.0043 (10)
N11A	0.0233 (11)	0.0483 (14)	0.0279 (12)	0.0001 (10)	0.0155 (9)	0.0043 (10)
C37A	0.0233 (11)	0.0483 (14)	0.0279 (12)	0.0001 (10)	0.0155 (9)	0.0043 (10)
C38A	0.0233 (11)	0.0483 (14)	0.0279 (12)	0.0001 (10)	0.0155 (9)	0.0043 (10)
C39A	0.0233 (11)	0.0483 (14)	0.0279 (12)	0.0001 (10)	0.0155 (9)	0.0043 (10)
C40A	0.0233 (11)	0.0483 (14)	0.0279 (12)	0.0001 (10)	0.0155 (9)	0.0043 (10)
C41A	0.0233 (11)	0.0483 (14)	0.0279 (12)	0.0001 (10)	0.0155 (9)	0.0043 (10)
C42A	0.0233 (11)	0.0483 (14)	0.0279 (12)	0.0001 (10)	0.0155 (9)	0.0043 (10)
O10	0.0415 (10)	0.0343 (9)	0.0229 (8)	0.0017 (7)	0.0169 (7)	0.0014 (7)
O11	0.0662 (13)	0.0292 (9)	0.0313 (9)	−0.0019 (8)	0.0301 (9)	−0.0056 (7)
O12	0.0511 (12)	0.0377 (10)	0.0504 (11)	−0.0157 (8)	0.0362 (10)	−0.0122 (8)
O13	0.0527 (12)	0.0428 (10)	0.0281 (9)	0.0101 (9)	0.0218 (9)	0.0101 (8)
O14	0.0400 (10)	0.0539 (11)	0.0276 (8)	−0.0025 (8)	0.0180 (8)	−0.0088 (8)
O15	0.0383 (11)	0.0397 (11)	0.0665 (14)	−0.0106 (8)	0.0268 (10)	0.0036 (9)
O16	0.0443 (11)	0.0332 (9)	0.0361 (9)	0.0053 (7)	0.0255 (9)	0.0098 (7)
N12	0.0353 (11)	0.0232 (9)	0.0306 (10)	0.0025 (8)	0.0200 (9)	−0.0019 (8)
N13	0.0299 (10)	0.0399 (11)	0.0242 (9)	0.0073 (8)	0.0159 (8)	0.0000 (8)
N14	0.0335 (11)	0.0276 (10)	0.0385 (11)	0.0003 (8)	0.0258 (9)	0.0009 (8)
C43	0.0248 (10)	0.0262 (10)	0.0259 (10)	0.0042 (8)	0.0170 (9)	0.0009 (8)
C44	0.0253 (10)	0.0228 (10)	0.0264 (10)	0.0015 (8)	0.0168 (9)	−0.0022 (8)
C45	0.0285 (11)	0.0243 (10)	0.0303 (11)	0.0060 (8)	0.0216 (9)	0.0034 (9)
C46	0.0250 (11)	0.0318 (11)	0.0215 (10)	0.0073 (8)	0.0150 (9)	0.0024 (8)
C47	0.0219 (10)	0.0280 (11)	0.0280 (11)	0.0010 (8)	0.0148 (9)	−0.0049 (9)
C48	0.0236 (10)	0.0232 (10)	0.0278 (10)	0.0021 (8)	0.0175 (9)	0.0025 (8)
N15	0.073 (2)	0.093 (3)	0.0564 (19)	−0.011 (2)	0.0263 (18)	−0.0007 (19)
C49	0.0415 (18)	0.109 (3)	0.072 (2)	−0.0059 (19)	0.0352 (18)	−0.033 (2)
C50	0.0366 (16)	0.098 (3)	0.0354 (15)	−0.0093 (17)	0.0180 (13)	−0.0140 (18)
N17	0.0259 (19)	0.030 (2)	0.057 (3)	0.0023 (16)	0.015 (2)	−0.0053 (18)
C51	0.0259 (19)	0.030 (2)	0.057 (3)	0.0023 (16)	0.015 (2)	−0.0053 (18)
C52	0.0259 (19)	0.030 (2)	0.057 (3)	0.0023 (16)	0.015 (2)	−0.0053 (18)
N17'	0.0259 (19)	0.030 (2)	0.057 (3)	0.0023 (16)	0.015 (2)	−0.0053 (18)
C51'	0.0259 (19)	0.030 (2)	0.057 (3)	0.0023 (16)	0.015 (2)	−0.0053 (18)
C52'	0.0259 (19)	0.030 (2)	0.057 (3)	0.0023 (16)	0.015 (2)	−0.0053 (18)
O1W	0.143 (12)	0.058 (7)	0.061 (7)	0.009 (7)	0.034 (7)	−0.006 (5)

### Geometric parameters (Å, °)

**Table:**

Cd—N5	2.2322 (17)	C31—H31A	0.9500
Cd—N1	2.2384 (18)	C32—C33	1.392 (3)
Cd—N7	2.2409 (17)	C32—H32A	0.9500
Cd—N3	2.2583 (18)	C33—C34	1.385 (3)
Cd—O1	2.4848 (16)	C33—H33A	0.9500
Cd—O2	2.5488 (16)	C35—H35A	0.9800
O1—C9	1.410 (3)	C35—H35B	0.9800
O1—C8	1.411 (3)	C35—H35C	0.9800
O2—C27	1.414 (3)	C36—H36A	0.9800
O2—C26	1.419 (3)	C36—H36B	0.9800
N1—C7	1.316 (3)	C36—H36C	0.9800
N1—C1	1.387 (3)	O3—C37	1.292 (5)
N2—C7	1.351 (3)	O4—N9	1.233 (6)
N2—C6	1.380 (4)	O5—N9	1.219 (6)
N2—C17	1.462 (3)	O6—N10	1.239 (7)
N3—C10	1.325 (3)	O7—N10	1.241 (6)
N3—C16	1.389 (3)	O8—N11	1.248 (6)
N4—C10	1.347 (3)	O9—N11	1.216 (7)
N4—C11	1.395 (3)	N9—C38	1.461 (5)
N4—C18	1.464 (3)	N10—C40	1.436 (5)
N5—C25	1.314 (3)	N11—C42	1.461 (4)
N5—C19	1.389 (3)	C37—C38	1.3900
N6—C25	1.357 (3)	C37—C42	1.3900
N6—C24	1.387 (3)	C38—C39	1.3900
N6—C35	1.457 (3)	C39—C40	1.3900
N7—C28	1.327 (3)	C39—H39	0.9500
N7—C34	1.396 (3)	C40—C41	1.3900
N8—C28	1.350 (3)	C41—C42	1.3900
N8—C29	1.385 (3)	C41—H41	0.9500
N8—C36	1.462 (3)	O3A—C37A	1.249 (5)
C1—C6	1.393 (3)	O4A—N9A	1.224 (6)
C1—C2	1.395 (4)	O5A—N9A	1.226 (7)
C2—C3	1.372 (4)	O6A—N10A	1.234 (7)
C2—H2A	0.9500	O7A—N10A	1.243 (6)
C3—C4	1.403 (5)	O8A—N11A	1.241 (6)
C3—H3A	0.9500	O9A—N11A	1.237 (7)
C4—C5	1.380 (5)	N9A—C38A	1.468 (5)
C4—H4A	0.9500	N10A—C40A	1.438 (5)
C5—C6	1.386 (4)	N11A—C42A	1.473 (6)
C5—H5A	0.9500	C37A—C42A	1.433 (6)
C7—C8	1.494 (3)	C37A—C38A	1.457 (6)
C8—H8A	0.9900	C38A—C39A	1.382 (5)
C8—H8B	0.9900	C39A—C40A	1.391 (6)
C9—C10	1.497 (3)	C39A—H39A	0.9500
C9—H9A	0.9900	C40A—C41A	1.400 (6)
C9—H9B	0.9900	C41A—C42A	1.385 (5)
C11—C12	1.388 (4)	C41A—H41A	0.9500
C11—C16	1.397 (3)	O10—C43	1.238 (3)
C12—C13	1.380 (4)	O11—N12	1.227 (3)
C12—H12A	0.9500	O12—N12	1.227 (3)
C13—C14	1.403 (4)	O13—N13	1.236 (3)
C13—H13A	0.9500	O14—N13	1.237 (3)
C14—C15	1.383 (3)	O15—N14	1.229 (3)
C14—H14A	0.9500	O16—N14	1.229 (3)
C15—C16	1.398 (3)	N12—C44	1.449 (3)
C15—H15A	0.9500	N13—C46	1.438 (3)
C17—H17A	0.9800	N14—C48	1.455 (3)
C17—H17B	0.9800	C43—C48	1.454 (3)
C17—H17C	0.9800	C43—C44	1.461 (3)
C18—H18A	0.9800	C44—C45	1.367 (3)
C18—H18B	0.9800	C45—C46	1.386 (3)
C18—H18C	0.9800	C45—H45	0.9500
C19—C20	1.390 (3)	C46—C47	1.393 (3)
C19—C24	1.399 (3)	C47—C48	1.373 (3)
C20—C21	1.381 (3)	C47—H47	0.9500
C20—H20A	0.9500	N15—C50	1.152 (5)
C21—C22	1.407 (3)	C49—C50	1.415 (5)
C21—H21A	0.9500	C49—H49A	0.9800
C22—C23	1.379 (3)	C49—H49B	0.9800
C22—H22A	0.9500	C49—H49C	0.9800
C23—C24	1.391 (3)	N17—C52	1.164 (6)
C23—H23A	0.9500	C51—C52	1.396 (6)
C25—C26	1.491 (3)	C51—H51A	0.9800
C26—H26A	0.9900	C51—H51B	0.9800
C26—H26B	0.9900	C51—H51C	0.9800
C27—C28	1.490 (3)	N17'—N17'^i^	0.795 (16)
C27—H27A	0.9900	N17'—C52'	1.158 (6)
C27—H27B	0.9900	N17'—C52'^i^	1.738 (13)
C29—C30	1.393 (3)	C51'—C52'	1.410 (6)
C29—C34	1.404 (3)	C51'—H51D	0.9800
C30—C31	1.386 (4)	C51'—H51E	0.9800
C30—H30A	0.9500	C51'—H51F	0.9800
C31—C32	1.394 (4)	C52'—N17'^i^	1.738 (13)
			
N5—Cd—N1	104.07 (7)	C28—C27—H27B	110.6
N5—Cd—N7	132.12 (6)	H27A—C27—H27B	108.8
N1—Cd—N7	96.09 (7)	N7—C28—N8	112.49 (18)
N5—Cd—N3	95.51 (6)	N7—C28—C27	123.78 (18)
N1—Cd—N3	132.44 (7)	N8—C28—C27	123.73 (18)
N7—Cd—N3	102.05 (6)	N8—C29—C30	132.6 (2)
N5—Cd—O1	113.26 (7)	N8—C29—C34	105.81 (18)
N1—Cd—O1	66.54 (6)	C30—C29—C34	121.6 (2)
N7—Cd—O1	114.58 (7)	C31—C30—C29	116.4 (2)
N3—Cd—O1	65.93 (6)	C31—C30—H30A	121.8
N5—Cd—O2	66.25 (6)	C29—C30—H30A	121.8
N1—Cd—O2	106.34 (7)	C30—C31—C32	122.3 (2)
N7—Cd—O2	66.52 (6)	C30—C31—H31A	118.9
N3—Cd—O2	121.21 (6)	C32—C31—H31A	118.9
O1—Cd—O2	172.75 (6)	C33—C32—C31	121.3 (2)
C9—O1—C8	116.64 (17)	C33—C32—H32A	119.4
C9—O1—Cd	122.27 (13)	C31—C32—H32A	119.4
C8—O1—Cd	120.73 (14)	C34—C33—C32	117.1 (2)
C27—O2—C26	116.02 (16)	C34—C33—H33A	121.5
C27—O2—Cd	118.42 (13)	C32—C33—H33A	121.5
C26—O2—Cd	118.45 (13)	C33—C34—N7	130.09 (19)
C7—N1—C1	105.72 (19)	C33—C34—C29	121.4 (2)
C7—N1—Cd	123.42 (16)	N7—C34—C29	108.52 (18)
C1—N1—Cd	130.20 (15)	N6—C35—H35A	109.5
C7—N2—C6	107.2 (2)	N6—C35—H35B	109.5
C7—N2—C17	126.7 (2)	H35A—C35—H35B	109.5
C6—N2—C17	126.1 (2)	N6—C35—H35C	109.5
C10—N3—C16	105.58 (18)	H35A—C35—H35C	109.5
C10—N3—Cd	124.19 (15)	H35B—C35—H35C	109.5
C16—N3—Cd	130.19 (14)	N8—C36—H36A	109.5
C10—N4—C11	106.83 (18)	N8—C36—H36B	109.5
C10—N4—C18	128.0 (2)	H36A—C36—H36B	109.5
C11—N4—C18	125.2 (2)	N8—C36—H36C	109.5
C25—N5—C19	106.11 (17)	H36A—C36—H36C	109.5
C25—N5—Cd	124.03 (14)	H36B—C36—H36C	109.5
C19—N5—Cd	129.33 (13)	O5—N9—O4	124.6 (8)
C25—N6—C24	106.95 (17)	O5—N9—C38	119.7 (7)
C25—N6—C35	126.3 (2)	O4—N9—C38	115.6 (6)
C24—N6—C35	126.73 (19)	O6—N10—O7	121.5 (9)
C28—N7—C34	105.79 (17)	O6—N10—C40	117.9 (8)
C28—N7—Cd	123.49 (14)	O7—N10—C40	119.6 (8)
C34—N7—Cd	130.41 (13)	O9—N11—O8	124.2 (7)
C28—N8—C29	107.38 (17)	O9—N11—C42	119.5 (7)
C28—N8—C36	125.80 (19)	O8—N11—C42	116.3 (6)
C29—N8—C36	126.78 (19)	O3—C37—C38	118.3 (4)
N1—C1—C6	109.0 (2)	O3—C37—C42	121.3 (4)
N1—C1—C2	130.3 (2)	C38—C37—C42	120.0
C6—C1—C2	120.7 (2)	C37—C38—C39	120.0
C3—C2—C1	117.4 (3)	C37—C38—N9	121.4 (3)
C3—C2—H2A	121.3	C39—C38—N9	118.4 (3)
C1—C2—H2A	121.3	C40—C39—C38	120.0
C2—C3—C4	121.3 (3)	C40—C39—H39	120.0
C2—C3—H3A	119.3	C38—C39—H39	120.0
C4—C3—H3A	119.3	C39—C40—C41	120.0
C5—C4—C3	121.8 (3)	C39—C40—N10	119.5 (4)
C5—C4—H4A	119.1	C41—C40—N10	120.0 (4)
C3—C4—H4A	119.1	C42—C41—C40	120.0
C4—C5—C6	116.5 (3)	C42—C41—H41	120.0
C4—C5—H5A	121.8	C40—C41—H41	120.0
C6—C5—H5A	121.8	C41—C42—C37	120.0
N2—C6—C5	132.1 (3)	C41—C42—N11	115.0 (3)
N2—C6—C1	105.6 (2)	C37—C42—N11	124.9 (3)
C5—C6—C1	122.3 (3)	O4A—N9A—O5A	124.8 (7)
N1—C7—N2	112.4 (2)	O4A—N9A—C38A	117.0 (6)
N1—C7—C8	123.2 (2)	O5A—N9A—C38A	118.0 (6)
N2—C7—C8	124.4 (2)	O6A—N10A—O7A	122.4 (9)
O1—C8—C7	104.70 (17)	O6A—N10A—C40A	119.4 (8)
O1—C8—H8A	110.8	O7A—N10A—C40A	117.2 (8)
C7—C8—H8A	110.8	O9A—N11A—O8A	124.6 (8)
O1—C8—H8B	110.8	O9A—N11A—C42A	114.7 (7)
C7—C8—H8B	110.8	O8A—N11A—C42A	120.5 (7)
H8A—C8—H8B	108.9	O3A—C37A—C42A	126.3 (5)
O1—C9—C10	105.19 (17)	O3A—C37A—C38A	122.5 (5)
O1—C9—H9A	110.7	C42A—C37A—C38A	111.2 (4)
C10—C9—H9A	110.7	C39A—C38A—C37A	126.3 (4)
O1—C9—H9B	110.7	C39A—C38A—N9A	115.9 (4)
C10—C9—H9B	110.7	C37A—C38A—N9A	117.6 (4)
H9A—C9—H9B	108.8	C38A—C39A—C40A	116.8 (4)
N3—C10—N4	112.90 (19)	C38A—C39A—H39A	121.6
N3—C10—C9	122.42 (19)	C40A—C39A—H39A	121.6
N4—C10—C9	124.68 (19)	C39A—C40A—C41A	121.8 (4)
C12—C11—N4	132.3 (2)	C39A—C40A—N10A	118.8 (5)
C12—C11—C16	122.1 (2)	C41A—C40A—N10A	119.0 (5)
N4—C11—C16	105.6 (2)	C42A—C41A—C40A	118.9 (4)
C13—C12—C11	116.4 (2)	C42A—C41A—H41A	120.5
C13—C12—H12A	121.8	C40A—C41A—H41A	120.5
C11—C12—H12A	121.8	C41A—C42A—C37A	124.4 (4)
C12—C13—C14	122.2 (3)	C41A—C42A—N11A	116.7 (4)
C12—C13—H13A	118.9	C37A—C42A—N11A	118.8 (4)
C14—C13—H13A	118.9	O12—N12—O11	123.0 (2)
C15—C14—C13	121.3 (2)	O12—N12—C44	118.10 (18)
C15—C14—H14A	119.3	O11—N12—C44	118.92 (19)
C13—C14—H14A	119.3	O13—N13—O14	123.1 (2)
C14—C15—C16	116.9 (2)	O13—N13—C46	118.8 (2)
C14—C15—H15A	121.6	O14—N13—C46	118.1 (2)
C16—C15—H15A	121.6	O15—N14—O16	123.2 (2)
N3—C16—C11	109.07 (19)	O15—N14—C48	118.1 (2)
N3—C16—C15	129.9 (2)	O16—N14—C48	118.69 (19)
C11—C16—C15	121.1 (2)	O10—C43—C48	125.1 (2)
N2—C17—H17A	109.5	O10—C43—C44	124.5 (2)
N2—C17—H17B	109.5	C48—C43—C44	110.40 (18)
H17A—C17—H17B	109.5	C45—C44—N12	117.46 (19)
N2—C17—H17C	109.5	C45—C44—C43	125.5 (2)
H17A—C17—H17C	109.5	N12—C44—C43	116.88 (19)
H17B—C17—H17C	109.5	C44—C45—C46	119.0 (2)
N4—C18—H18A	109.5	C44—C45—H45	120.5
N4—C18—H18B	109.5	C46—C45—H45	120.5
H18A—C18—H18B	109.5	C45—C46—C47	120.9 (2)
N4—C18—H18C	109.5	C45—C46—N13	119.0 (2)
H18A—C18—H18C	109.5	C47—C46—N13	120.1 (2)
H18B—C18—H18C	109.5	C48—C47—C46	119.1 (2)
N5—C19—C20	130.28 (19)	C48—C47—H47	120.4
N5—C19—C24	108.76 (18)	C46—C47—H47	120.4
C20—C19—C24	121.0 (2)	C47—C48—C43	125.1 (2)
C21—C20—C19	117.3 (2)	C47—C48—N14	116.7 (2)
C21—C20—H20A	121.4	C43—C48—N14	118.22 (19)
C19—C20—H20A	121.4	C50—C49—H49A	109.5
C20—C21—C22	121.2 (2)	C50—C49—H49B	109.5
C20—C21—H21A	119.4	H49A—C49—H49B	109.5
C22—C21—H21A	119.4	C50—C49—H49C	109.5
C23—C22—C21	122.1 (2)	H49A—C49—H49C	109.5
C23—C22—H22A	119.0	H49B—C49—H49C	109.5
C21—C22—H22A	119.0	N15—C50—C49	179.3 (5)
C22—C23—C24	116.3 (2)	C52—C51—H51A	109.5
C22—C23—H23A	121.8	C52—C51—H51B	109.5
C24—C23—H23A	121.8	H51A—C51—H51B	109.5
N6—C24—C23	132.2 (2)	C52—C51—H51C	109.5
N6—C24—C19	105.67 (18)	H51A—C51—H51C	109.5
C23—C24—C19	122.1 (2)	H51B—C51—H51C	109.5
N5—C25—N6	112.50 (19)	N17—C52—C51	176.2 (13)
N5—C25—C26	124.00 (18)	N17'^i^—N17'—C52'	124.6 (10)
N6—C25—C26	123.50 (18)	N17'^i^—N17'—C52'^i^	33.3 (6)
O2—C26—C25	104.78 (16)	C52'—N17'—C52'^i^	102.4 (11)
O2—C26—H26A	110.8	C52'—C51'—H51D	109.5
C25—C26—H26A	110.8	C52'—C51'—H51E	109.5
O2—C26—H26B	110.8	H51D—C51'—H51E	109.5
C25—C26—H26B	110.8	C52'—C51'—H51F	109.5
H26A—C26—H26B	108.9	H51D—C51'—H51F	109.5
O2—C27—C28	105.58 (16)	H51E—C51'—H51F	109.5
O2—C27—H27A	110.6	N17'—C52'—C51'	172.8 (12)
C28—C27—H27A	110.6	N17'—C52'—N17'^i^	22.1 (5)
O2—C27—H27B	110.6	C51'—C52'—N17'^i^	150.8 (10)
			
N5—Cd—O1—C9	85.08 (19)	C22—C23—C24—C19	−0.8 (3)
N1—Cd—O1—C9	−179.1 (2)	N5—C19—C24—N6	0.3 (2)
N7—Cd—O1—C9	−93.16 (19)	C20—C19—C24—N6	−179.8 (2)
N3—Cd—O1—C9	−0.47 (17)	N5—C19—C24—C23	−179.0 (2)
O2—Cd—O1—C9	169.7 (4)	C20—C19—C24—C23	0.9 (3)
N5—Cd—O1—C8	−87.77 (19)	C19—N5—C25—N6	0.1 (2)
N1—Cd—O1—C8	8.07 (17)	Cd—N5—C25—N6	−172.28 (13)
N7—Cd—O1—C8	93.98 (19)	C19—N5—C25—C26	180.0 (2)
N3—Cd—O1—C8	−173.3 (2)	Cd—N5—C25—C26	7.6 (3)
O2—Cd—O1—C8	−3.2 (6)	C24—N6—C25—N5	0.1 (2)
N5—Cd—O2—C27	−160.70 (18)	C35—N6—C25—N5	178.7 (2)
N1—Cd—O2—C27	100.81 (17)	C24—N6—C25—C26	−179.78 (19)
N7—Cd—O2—C27	11.20 (15)	C35—N6—C25—C26	−1.2 (3)
N3—Cd—O2—C27	−78.97 (17)	C27—O2—C26—C25	166.71 (19)
O1—Cd—O2—C27	111.6 (5)	Cd—O2—C26—C25	16.6 (2)
N5—Cd—O2—C26	−11.34 (15)	N5—C25—C26—O2	−16.2 (3)
N1—Cd—O2—C26	−109.84 (16)	N6—C25—C26—O2	163.7 (2)
N7—Cd—O2—C26	160.55 (18)	C26—O2—C27—C28	−166.03 (19)
N3—Cd—O2—C26	70.39 (17)	Cd—O2—C27—C28	−15.9 (2)
O1—Cd—O2—C26	−99.1 (5)	C34—N7—C28—N8	0.0 (2)
N5—Cd—N1—C7	109.26 (18)	Cd—N7—C28—N8	174.22 (13)
N7—Cd—N1—C7	−114.51 (18)	C34—N7—C28—C27	179.87 (19)
N3—Cd—N1—C7	−2.1 (2)	Cd—N7—C28—C27	−6.0 (3)
O1—Cd—N1—C7	−0.32 (17)	C29—N8—C28—N7	0.6 (2)
O2—Cd—N1—C7	178.21 (17)	C36—N8—C28—N7	178.5 (2)
N5—Cd—N1—C1	−81.4 (2)	C29—N8—C28—C27	−179.2 (2)
N7—Cd—N1—C1	54.9 (2)	C36—N8—C28—C27	−1.4 (3)
N3—Cd—N1—C1	167.32 (19)	O2—C27—C28—N7	14.7 (3)
O1—Cd—N1—C1	169.1 (2)	O2—C27—C28—N8	−165.5 (2)
O2—Cd—N1—C1	−12.4 (2)	C28—N8—C29—C30	177.6 (2)
N5—Cd—N3—C10	−111.97 (16)	C36—N8—C29—C30	−0.3 (4)
N1—Cd—N3—C10	2.8 (2)	C28—N8—C29—C34	−1.0 (2)
N7—Cd—N3—C10	112.83 (16)	C36—N8—C29—C34	−178.8 (2)
O1—Cd—N3—C10	1.08 (16)	N8—C29—C30—C31	−178.9 (2)
O2—Cd—N3—C10	−177.47 (15)	C34—C29—C30—C31	−0.6 (3)
N5—Cd—N3—C16	65.11 (18)	C29—C30—C31—C32	−0.6 (4)
N1—Cd—N3—C16	179.90 (16)	C30—C31—C32—C33	0.8 (4)
N7—Cd—N3—C16	−70.09 (18)	C31—C32—C33—C34	0.1 (3)
O1—Cd—N3—C16	178.16 (19)	C32—C33—C34—N7	177.8 (2)
O2—Cd—N3—C16	−0.39 (19)	C32—C33—C34—C29	−1.2 (3)
N1—Cd—N5—C25	103.81 (17)	C28—N7—C34—C33	−179.8 (2)
N7—Cd—N5—C25	−8.2 (2)	Cd—N7—C34—C33	6.6 (3)
N3—Cd—N5—C25	−119.88 (17)	C28—N7—C34—C29	−0.7 (2)
O1—Cd—N5—C25	173.99 (15)	Cd—N7—C34—C29	−174.29 (14)
O2—Cd—N5—C25	1.88 (16)	N8—C29—C34—C33	−179.75 (19)
N1—Cd—N5—C19	−66.64 (18)	C30—C29—C34—C33	1.5 (3)
N7—Cd—N5—C19	−178.62 (15)	N8—C29—C34—N7	1.0 (2)
N3—Cd—N5—C19	69.67 (18)	C30—C29—C34—N7	−177.7 (2)
O1—Cd—N5—C19	3.54 (19)	O3—C37—C38—C39	−173.0 (5)
O2—Cd—N5—C19	−168.57 (19)	C42—C37—C38—C39	0.0
N5—Cd—N7—C28	7.4 (2)	O3—C37—C38—N9	12.7 (6)
N1—Cd—N7—C28	−107.82 (16)	C42—C37—C38—N9	−174.2 (4)
N3—Cd—N7—C28	116.39 (16)	O5—N9—C38—C37	−130.3 (12)
O1—Cd—N7—C28	−174.77 (15)	O4—N9—C38—C37	46.1 (11)
O2—Cd—N7—C28	−2.62 (15)	O5—N9—C38—C39	55.4 (13)
N5—Cd—N7—C34	−179.96 (15)	O4—N9—C38—C39	−128.3 (10)
N1—Cd—N7—C34	64.82 (18)	C37—C38—C39—C40	0.0
N3—Cd—N7—C34	−70.97 (18)	N9—C38—C39—C40	174.4 (4)
O1—Cd—N7—C34	−2.13 (19)	C38—C39—C40—C41	0.0
O2—Cd—N7—C34	170.02 (19)	C38—C39—C40—N10	171.8 (7)
C7—N1—C1—C6	−0.2 (3)	O6—N10—C40—C39	−1(2)
Cd—N1—C1—C6	−171.05 (19)	O7—N10—C40—C39	−170.0 (19)
C7—N1—C1—C2	178.8 (3)	O6—N10—C40—C41	170 (2)
Cd—N1—C1—C2	8.0 (4)	O7—N10—C40—C41	2(2)
N1—C1—C2—C3	−178.5 (3)	C39—C40—C41—C42	0.0
C6—C1—C2—C3	0.5 (5)	N10—C40—C41—C42	−171.7 (7)
C1—C2—C3—C4	0.8 (6)	C40—C41—C42—C37	0.0
C2—C3—C4—C5	−1.3 (8)	C40—C41—C42—N11	−175.5 (4)
C3—C4—C5—C6	0.5 (8)	O3—C37—C42—C41	172.8 (5)
C7—N2—C6—C5	−177.3 (4)	C38—C37—C42—C41	0.0
C17—N2—C6—C5	4.6 (6)	O3—C37—C42—N11	−12.1 (5)
C7—N2—C6—C1	0.4 (3)	C38—C37—C42—N11	175.1 (4)
C17—N2—C6—C1	−177.7 (3)	O9—N11—C42—C41	−162.4 (9)
C4—C5—C6—N2	178.1 (4)	O8—N11—C42—C41	19.1 (10)
C4—C5—C6—C1	0.8 (6)	O9—N11—C42—C37	22.3 (10)
N1—C1—C6—N2	−0.1 (3)	O8—N11—C42—C37	−156.2 (9)
C2—C1—C6—N2	−179.2 (3)	O3A—C37A—C38A—C39A	−176.0 (8)
N1—C1—C6—C5	177.9 (3)	C42A—C37A—C38A—C39A	4.5 (8)
C2—C1—C6—C5	−1.3 (5)	O3A—C37A—C38A—N9A	0.4 (9)
C1—N1—C7—N2	0.5 (3)	C42A—C37A—C38A—N9A	−179.1 (4)
Cd—N1—C7—N2	172.08 (16)	O4A—N9A—C38A—C39A	−132.7 (11)
C1—N1—C7—C8	−178.8 (2)	O5A—N9A—C38A—C39A	43.2 (14)
Cd—N1—C7—C8	−7.2 (3)	O4A—N9A—C38A—C37A	50.5 (11)
C6—N2—C7—N1	−0.6 (3)	O5A—N9A—C38A—C37A	−133.6 (12)
C17—N2—C7—N1	177.5 (3)	C37A—C38A—C39A—C40A	−5.6 (8)
C6—N2—C7—C8	178.7 (2)	N9A—C38A—C39A—C40A	177.9 (5)
C17—N2—C7—C8	−3.2 (4)	C38A—C39A—C40A—C41A	0.4 (7)
C9—O1—C8—C7	174.2 (2)	C38A—C39A—C40A—N10A	−172.1 (8)
Cd—O1—C8—C7	−12.6 (2)	O6A—N10A—C40A—C39A	4(2)
N1—C7—C8—O1	12.7 (3)	O7A—N10A—C40A—C39A	173.4 (19)
N2—C7—C8—O1	−166.5 (2)	O6A—N10A—C40A—C41A	−169 (2)
C8—O1—C9—C10	173.1 (2)	O7A—N10A—C40A—C41A	1(2)
Cd—O1—C9—C10	−0.1 (2)	C39A—C40A—C41A—C42A	5.4 (7)
C16—N3—C10—N4	0.2 (2)	N10A—C40A—C41A—C42A	177.9 (7)
Cd—N3—C10—N4	177.88 (13)	C40A—C41A—C42A—C37A	−6.6 (8)
C16—N3—C10—C9	−179.36 (19)	C40A—C41A—C42A—N11A	175.2 (5)
Cd—N3—C10—C9	−1.7 (3)	O3A—C37A—C42A—C41A	−177.6 (8)
C11—N4—C10—N3	−0.4 (2)	C38A—C37A—C42A—C41A	1.9 (7)
C18—N4—C10—N3	178.1 (2)	O3A—C37A—C42A—N11A	0.5 (9)
C11—N4—C10—C9	179.1 (2)	C38A—C37A—C42A—N11A	−180.0 (4)
C18—N4—C10—C9	−2.4 (4)	O9A—N11A—C42A—C41A	−153.8 (10)
O1—C9—C10—N3	1.0 (3)	O8A—N11A—C42A—C41A	21.3 (13)
O1—C9—C10—N4	−178.5 (2)	O9A—N11A—C42A—C37A	28.0 (11)
C10—N4—C11—C12	−179.8 (3)	O8A—N11A—C42A—C37A	−156.9 (12)
C18—N4—C11—C12	1.6 (4)	O12—N12—C44—C45	36.4 (3)
C10—N4—C11—C16	0.5 (2)	O11—N12—C44—C45	−144.4 (2)
C18—N4—C11—C16	−178.1 (2)	O12—N12—C44—C43	−138.9 (2)
N4—C11—C12—C13	−179.9 (3)	O11—N12—C44—C43	40.3 (3)
C16—C11—C12—C13	−0.2 (4)	O10—C43—C44—C45	−178.6 (2)
C11—C12—C13—C14	0.9 (4)	C48—C43—C44—C45	0.9 (3)
C12—C13—C14—C15	−0.3 (5)	O10—C43—C44—N12	−3.7 (3)
C13—C14—C15—C16	−0.9 (4)	C48—C43—C44—N12	175.77 (17)
C10—N3—C16—C11	0.1 (2)	N12—C44—C45—C46	−176.88 (18)
Cd—N3—C16—C11	−177.37 (14)	C43—C44—C45—C46	−2.0 (3)
C10—N3—C16—C15	−178.9 (2)	C44—C45—C46—C47	0.9 (3)
Cd—N3—C16—C15	3.6 (3)	C44—C45—C46—N13	178.27 (19)
C12—C11—C16—N3	179.9 (2)	O13—N13—C46—C45	1.0 (3)
N4—C11—C16—N3	−0.4 (2)	O14—N13—C46—C45	−177.9 (2)
C12—C11—C16—C15	−1.0 (3)	O13—N13—C46—C47	178.4 (2)
N4—C11—C16—C15	178.8 (2)	O14—N13—C46—C47	−0.5 (3)
C14—C15—C16—N3	−179.6 (2)	C45—C46—C47—C48	1.3 (3)
C14—C15—C16—C11	1.5 (3)	N13—C46—C47—C48	−176.10 (19)
C25—N5—C19—C20	179.9 (2)	C46—C47—C48—C43	−2.5 (3)
Cd—N5—C19—C20	−8.3 (3)	C46—C47—C48—N14	175.21 (19)
C25—N5—C19—C24	−0.2 (2)	O10—C43—C48—C47	−179.1 (2)
Cd—N5—C19—C24	171.55 (14)	C44—C43—C48—C47	1.4 (3)
N5—C19—C20—C21	179.8 (2)	O10—C43—C48—N14	3.2 (3)
C24—C19—C20—C21	−0.1 (3)	C44—C43—C48—N14	−176.24 (18)
C19—C20—C21—C22	−0.7 (4)	O15—N14—C48—C47	33.0 (3)
C20—C21—C22—C23	0.8 (4)	O16—N14—C48—C47	−145.0 (2)
C21—C22—C23—C24	0.0 (4)	O15—N14—C48—C43	−149.1 (2)
C25—N6—C24—C23	179.0 (2)	O16—N14—C48—C43	32.9 (3)
C35—N6—C24—C23	0.4 (4)	N17'^i^—N17'—C52'—C51'	−13 (11)
C25—N6—C24—C19	−0.3 (2)	C52'^i^—N17'—C52'—C51'	14 (10)
C35—N6—C24—C19	−178.8 (2)	C52'^i^—N17'—C52'—N17'^i^	27.3 (15)
C22—C23—C24—N6	−180.0 (2)		

Symmetry codes: (i) −*x*, *y*, −*z*−1/2.
